# Disentangling the spatial and temporal causes of decline in a bird population

**DOI:** 10.1002/ece3.6244

**Published:** 2020-06-30

**Authors:** Floriane Plard, Raphaël Arlettaz, Alain Jacot, Michael Schaub

**Affiliations:** ^1^ Swiss Ornithological Institute Sempach Switzerland; ^2^ Division of Conservation Biology Institute of Ecology and Evolution University of Bern Bern Switzerland; ^3^ Swiss Ornithological Institute Sion Switzerland

**Keywords:** environmental influence, fledgling success, integrated population model, productivity, survival, *Upupa epops*

## Abstract

The difficulties in understanding the underlying reasons of a population decline lie in the typical short duration of field studies, the often too small size already reached by a declining population or the multitude of environmental factors that may influence population trend. In this difficult context, useful demographic tools such as integrated population models (IPM) may help disentangling the main reasons for a population decline. To understand why a hoopoe *Upupa epops* population has declined, we followed a three step model analysis. We built an IPM structured with respect to habitat quality (approximated by the expected availability of mole crickets, the main prey in our population) and estimated the contributions of habitat‐specific demographic rates to population variation and decline. We quantified how much each demographic rate has decreased and investigated whether habitat quality influenced this decline. We tested how much weather conditions and research activities contributed to the decrease in the different demographic rates. The decline of the hoopoe population was mainly explained by a decrease in first‐year apparent survival and a reduced number of fledglings produced, particularly in habitats of high quality. Since a majority of pairs bred in habitats of the highest quality, the decrease in the production of locally recruited yearlings in high‐quality habitat was the main driver of the population decline despite a homogeneous drop of recruitment across habitats. Overall, the explanatory variables we tested only accounted for 19% of the decrease in the population growth rate. Among these variables, the effects of spring temperature (49% of the explained variance) contributed more to population decline than spring precipitation (36%) and research activities (maternal capture delay, 15%). This study shows the power of IPMs for identifying the vital rates involved in population declines and thus paves the way for targeted conservation and management actions.

## Graphical abstract

This study disentangles spatial and temporal reasons of the decline of rare European bird population. It shows how demographic drivers as well as environmental factors responsible for a decline can be evidenced using an IPM structured by habitat quality and could be applied in many declining populations.

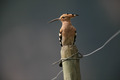

## 1 INTRODUCTION

The causes of a population decline are difficult to unravel (Caughley & Sinclair, 1994) since population dynamics can be influenced by multiple factors acting on different scales (Coulson et al., 2001; Molnár, Derocher, Thiemann, & Lewis, 2010). The difficulty arises due to the often complex interactions between intrinsic and environmental factors that lead to weak or spurious correlations with population density trends (Hayes & Steidl, 1997), short study duration, or small population size. There is a multitude of factors impacting population abundance. Thus, simple correlations between abundance and isolated factors are often hard to interpret. Among environmental factors, habitat degradation and loss, and environmental pollution caused by general anthropogenic perturbation as well as weather variability have often been evidenced as main factors negatively impacting population size (Parmesan, 2006). Other anthropogenic disturbance such as outdoor recreation or research activities can additionally negatively affect population trends (Arlettaz et al., 2015; Schaub & Abadi, 2011; Tablado & Jenni, 2017), but their influence is rarely investigated.

Different approaches can be combined to identify the possible reasons for a population decline. The first one is the decomposition of the temporal variability of population growth into the contribution of the variability of the underlying demographic rates. This approach allows the identification of the demographic processes that have been most strongly driving the dynamics of a population (Ehrlén & van Groenendael, 1998). In a next step, it can be investigated through which environmental processes these identified demographic variables were affected. A second approach is to include spatial in addition to temporal variability (Rushing et al., 2017). Individuals typically inhabit habitats that differ in quality due to differential availability of breeding and food resources (Pulliam, 1988; Johnson, 2007). Heterogeneity in habitat quality can affect demography. For instance, blue tits (*Cyanistes caeruleus*) living in rich broad‐leaved deciduous forest patches produced more fledglings than those living in poor patches (Lambrechts et al., 2004). The combination of temporal and spatial approaches allows understanding as to whether demographic processes linked to population decline are consistent among habitats of different qualities and to identify whether some habitats should be preserved or restored in priority to slow down or halt population decline.

Integrated population models (IPM) are powerful tools to estimate demographic rates and population size (Besbeas, Freeman, Morgan, & Catchpole, 2002; Schaub & Abadi, 2011). Different data sets such as productivity, capture–recapture, and population count data are jointly analyzed in IPM with the advantages to estimate demographic parameters more precisely and to allow the estimation of demographic rates for which no explicit data have been collected (Abadi, Gimenez, Arlettaz, & Schaub, 2010). For instance, the effect of a variable influencing wind conditions on juvenile survival in the emperor penguins (*Aptenodytes forsteri*), a demographic rate for which no direct data were available, was revealed by an IPM analysis (Abadi, Barbraud, & Gimenez, 2017). Results from an IPM can then be used to investigate the contribution of each demographic rate and of population structure onto the variation of the population growth rate over the study period and to the difference of mean population growth rates between periods of time (Koons, Arnold, & Schaub, 2017; Koons, Iles, Schaub, & Caswell, 2016). Finally, IPM can be spatially structured to investigate the influence of habitat quality on population dynamics (McCrea et al., 2010; Péron, Crochet, Doherty, & Lebreton, 2010). Using this approach, Rushing et al. (2017) showed that population growth of Wood Thrush (*Hylocichla mustelina*) was sensitive to juvenile survival in high‐quality habitats and to adult survival in low‐quality habitats.

Here, we develop an IPM structured by habitat quality to study the recent decline of a population of the European hoopoe *Upupa epops*. This species has declined in Central and Western Europe over the last century (Arlettaz, Schaub, et al., 2010) and is listed as endangered on the Red List of endangered species of Switzerland (Keller, Gerber, Schmid, Volet, & Zbinden, 2010). The largest Swiss population is located in Valais (Knaus et al., 2018), which is our study area. This population declined in the 1980' and 1990' to the point that the hoopoe almost went extinct locally (Arlettaz, Schaub, et al., 2010). The intensification of human activities such as agriculture and urbanization has caused a quasi‐disappearance of natural cavities on which hoopoes relied for breeding. From 1999 to 2003, more than 700 nest boxes were installed in the study area as a mitigation measure. The number of broods increased sixfold in the following years, but has been in steady decline since 2006, that is, just after reaching its demographic peak (Figure 2). Our goal was to identify demographic drivers and environmental variables that were responsible for the observed changes in population dynamics since 2002 when a rigorous capture–mark–recapture monitoring protocol started. We followed a three step analysis to reach this goal. We first quantified the contribution of each habitat‐specific demographic rate to the variation in the population growth rate and to the decline of the population. Second, we investigated whether these demographic rates have dropped over the study period and if so, whether it was consistent among habitats. Third, we tested the influence of weather conditions and of research activity on these demographic rates to better understand the potential mechanisms that have led to the decline of the hoopoe population. These results were finally put into context, many aspects of the ecology of the local hoopoe population being known, notably in relation to trophic and foraging considerations.

## 2 METHODS

### 2.1 Studied population

**Figure 1:**
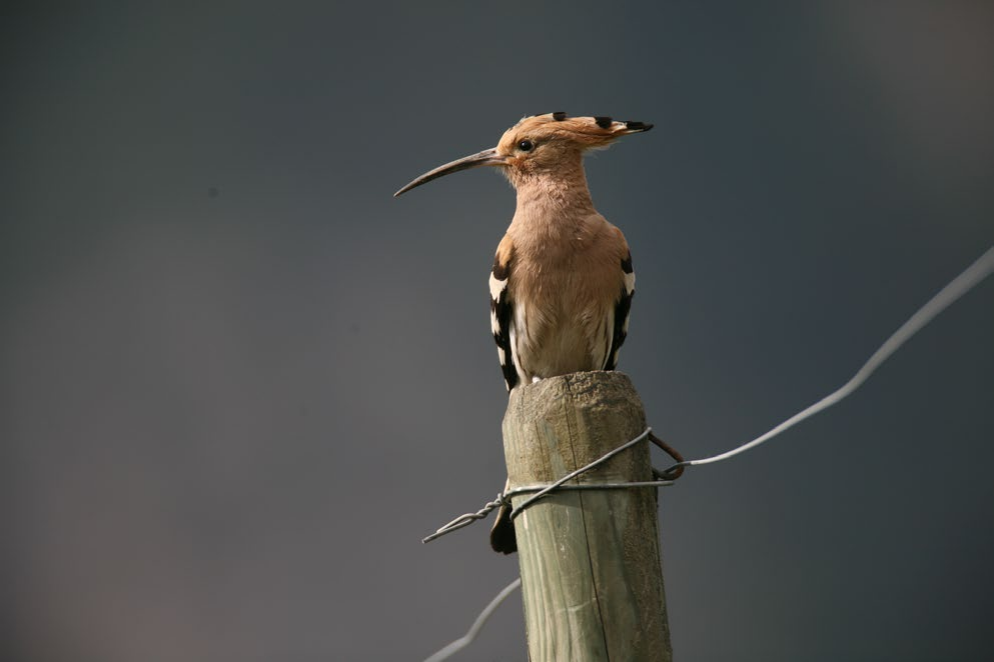
Hoopoe from the population in the Valais, Switzerland, studied from 2002 to 2017. credits: R. Arlettaz.

The hoopoe (Figure 1) is a long‐distance migrant bird breeding in Europe and wintering in Western Africa (van Wijk et al., 2018). Hoopoes occur in open landscapes, breed in natural and artificial cavities, and mostly feed on large ground‐dwelling insects (Martín‐Vivaldi, Palomino, Soler, & Soler, 1999). Hoopoes are short‐lived and highly productive, being able to regularly raise two broods in a breeding season (Hoffmann, Postma, & Schaub, 2015; Schaub et al., 2012).

The study area is located on the plain of the Upper Rhône valley, south‐western Swiss Alps (46°140N, 7°22E, 460–520 m altitude, 64 km^2^), where intensive farming, mostly of dwarf fruit tree plantations, vegetable crops, and vineyards dominate. The study population has been surveyed with intensive mark–recapture efforts every year from 2002 to 2017. All nest boxes have been monitored every second week during the breeding season (April to August). Occupied boxes (less than a third of the boxes in most years) were visited every third to fifth day to record clutch size, hatching date, and fledgling success. Adults were captured either outside the nest box using mist nets or claptraps, or inside the nest box by hand. Captured individuals were ringed, aged based on molt, sexed based on the conspicuous uropygial gland, and morphological measures were taken (Martín‐Vivaldi et al., 2009; Plard, Schindler, Arlettaz, & Schaub, 2018; Schaub et al., 2012). The nestlings were ringed and morphologically measured when they were about 10 days old. Besides this standard procedure, some individuals were additionally tagged with transmitters (VHS‐Very High Frequency, geolocators or GPS‐Global Positioning System) in some years (Tagmann‐Ioset, Schaub, Reichlin, Weisshaupt, & Arlettaz, 2012; van Wijk, Bauer, & Schaub, 2016), and blood, feather, and/or claw samples were taken (Reichlin et al., 2013; Schmid et al., 2013).

### 2.2 Population model

To describe the hoopoe population, we constructed a model with three stage classes (local recruits ( $N_{t}^{1}$ ), experienced adult breeders ( $N_{t}^{2}$ ), and immigrants ( $N_{t}^{i}$ )). We used a prebreeding census, meaning that the population is modeled each year just before the hatching period. We used a population model based on females because males and females contributed almost equally to population growth and a population model that is restricted to one sex did not influence demographic rates of the population model (Plard et al., 2018; Schaub et al., 2012). The model assumed that apparent first‐year survival (*S^1^*) was different from apparent adult survival (*S^2^*), but reproductive (*R*) and immigration ( ω ) rates were assumed to be independent of age (Schaub et al., 2012). Permanent emigration rate was included in the estimate of annual apparent survival as it cannot be distinguished from mortality. The population model excluding spatial heterogeneity was as follows:

$$N_{t+1}^{1}∼Pois\left(R_{t}S_{t}^{1}N_{t}^{\mathrm{tot}}\right)$$

$$N_{t+1}^{2}∼Bin\left(S_{t}^{2},N_{t}^{\mathrm{tot}}\right)$$

$$N_{t+1}^{i}∼Pois\left(\omega_{t}N_{t}^{\mathrm{tot}}\right)$$

(1) $$N_{t+1}^{\mathrm{tot}}=N_{t+1}^{2}+N_{t+1}^{1}+N_{t+1}^{i}$$

We analyzed individual capture–recapture histories (*N* = 7,418 individuals) with a multistate capture–recapture model to estimate apparent annual survival ( $S_{t}^{1}$ , $S_{t}^{2}$ ), recapture, and movement probabilities (see IPM stratified by habitats). Based on a previous analysis of this population (Schaub et al., 2012), we included age (first‐year vs. after first‐year) but no sex effects on survival, movement, and recapture probabilities.

Annual productivity (*R_t_*) was defined as the total number of fledglings raised per female and year including double broods. We decomposed annual productivity into the probability of successful reproduction (probability of raising at least one fledgling within a breeding season) and the total number of fledglings raised by a successful female during the complete breeding season. When a female had two broods, the number of fledglings was thus the sum of the outcomes of the two broods (Figure S1). It is justified to use the annual productivity per female rather than per brood because the former accounts for possible replacement clutches. However, as the mother identity needed to be known, we excluded broods where the mother identity was unknown which were mostly broods that failed very early (*N* = 205). Because unsuccessful mothers can be captured when they laid replacement clutches, we probably overestimated the probability of successful reproduction only slightly. The probability of successful reproduction was modeled using the binomial distribution and the logit link function (*N* = 1,098). The number of fledglings raised by a successful female was modeled using the gamma distribution (*N* = 1,029). We multiplied annual reproductive success by 0.5 to keep only females in the model, assuming a balanced birth sex ratio (Schaub et al., 2012).

The maximum annual number of simultaneous active broods was used as an index of population size. The high recapture probability of individuals breeding in our population (81[75–86]%) indicates that the amount of nonbreeders is low and is unlikely to influence the population dynamics significantly. The observation process of the IPM linking the predicted population size ( $N_{t}^{\mathrm{tot}}$ ) to the index of population size was modeled with the Poisson distribution. The likelihood of the integrated population model was based on the likelihoods of the four data sets (state‐space model for population size index, multistate capture–recapture model for capture–recapture data, and generalized linear models for the two components of productivity).

### 2.3 IPM stratified by habitat quality

The above described IPM was stratified spatially according to habitat quality. Mole crickets (*Gryllotalpa gryllotalpa*) are the main prey for raising nestlings in the study area (93% of food biomass are mole crickets (Arlettaz, Schaad, Reichlin, & Schaub, 2010)). Parents that delivered a larger proportion of mole crickets produced more fledglings in better condition than parents feeding a lower proportion of this profitable prey (Guillod, Arlettaz, & Jacot, 2016). The occurrence of mole crickets is spatially heterogenous. This insect lives in self‐dug soil cavities, prefers humid and soft grounds (Tagmann‐Ioset et al., 2012), and its occurrence increases with increasing levels of ground water table (Tschumi, Schaub, & Arlettaz, 2014). We therefore considered territories in areas of high ground water table to be of higher quality than territories in areas of lower ground water table. We calculated the average ground water depth (publicly available data from the Département des Transports de lˊEquipement et de lˊEnvironnement, Etat du Valais et Centre de Recherche sur lˊEnvironnement Alpin—CREALP) in a radius of 300 m around each nest box (corresponds to an average territory size, (Tagmann‐Ioset et al., 2012)) and allocated them into three groups of equal size characterized with high, medium, and low ground water table. We labeled these groups as habitats of high (H), medium (M), and low (L) quality.

Because the nest boxes have been split such that the number of available nest boxes is identical in each type of habitat quality, the population sizes in each habitat (Figure 3) are thus directly comparable in terms of relative densities regarding availability of breeding sites between the different habitats. Breeding sites have been shown to be the main missing resource for hoopoes locally (Arlettaz, Schaub, et al., 2010) and thus are a good proxy of territory availability for this species.

We formulated an IPM in which different demographic rates for each level of habitat quality were estimated. The probabilities of movement (ψ) between habitats of different quality within the study area were estimated using a multistate capture–recapture model (Lebreton & Pradel, 2002). These probabilities differ from the immigration rate that estimates the proportion of individuals entering the study population, thus immigrants originate from outside the study area. We estimated distinct probabilities of movement for first‐year ( $\Psi^{1}$ ) and older individuals ( $\Psi^{2}$ ) as natal dispersal is typically stronger than breeding dispersal in birds (Greenwood & Harvey, 1982). In all models, probabilities of recapture included a random effect of year and were independent of the level of habitat quality where the birds were captured. The population model for individuals in high‐quality habitats (H) was as follows:

$$N_{H,t+1}^{1}∼Pois\left(R_{H,t}S_{H,t}^{1}\Psi_{\mathrm{HH}}^{1}N_{H,t}^{\mathrm{tot}}+R_{M,t}S_{M,t}^{1}\Psi_{\mathrm{MH}}^{1}N_{M,t}^{\mathrm{tot}}+R_{L,t}S_{L,t}^{1}\Psi_{\mathrm{LH}}^{1}N_{L,t}^{\mathrm{tot}}\right)$$

$$N_{H,t+1}^{2}∼Bin\left(S_{H,t}^{2}\Psi_{\mathrm{HH}}^{2},N_{H,t}^{\mathrm{tot}}\right)+Bin\left(S_{M,t}^{2}\Psi_{\mathrm{MH}}^{2},N_{M,t}^{\mathrm{tot}}\right)+Bin\left(S_{L,t}^{2}\Psi_{\mathrm{LH}}^{2},N_{L,t}^{\mathrm{tot}}\right)$$

$$N_{H,t+1}^{i}∼Pois\left(\omega_{H,t}N_{t}^{H,tot}\right)$$

(2) $$N_{H,t+1}^{\mathrm{tot}}=N_{H,t+1}^{2}+N_{H,t+1}^{1}+N_{H,t+1}^{i}$$

Similar population models were formulated for birds living in habitats with medium and low quality.

### 2.4 Analysis

To understand how much each demographic rate has contributed to the decline of the hoopoe population size, we built three different types of models (I, II, and III, Table 1). They differed in the assumption about annual variation in demographic rates. We applied the Bayesian framework to build the different models using JAGS (Plummer, 2003) run from R (R Core Team, 2014) via package jagsUI (Kellner, 2015). We specified vague priors such that they did not influence posterior distributions. We used normal distributions with mean 0 and variance 1,000 for intercepts and slopes except the intercept for the number of fledglings of a successful female for which we used a uniform distribution over the interval [0.01:15]. Uniform distributions over the interval [0:10] were used as prior distributions for standard deviations of random effects. We ran 3 Monte Carlo Markov chains of 3,000 iterations and used the first 1,500 as burn‐in and checked convergence of all parameters with the Rubin and Gelman convergence diagnostic (R < 1.01, (Gelman & Rubin, 1992)). We next describe the models of our 3‐steps approach.

**Table 1:** Description of the three types of models. Model I uses year as a random effect; purpose: assess the contribution of the variability of habitat‐specific demographic rates on the variability of the population growth rate. Model II used year as a continuous variable; purpose: investigate habitat‐specific trends in demographic rates. Model III included additional variables (weather, research activity); purpose: testing specific hypotheses. 1 represents a model with an intercept only. Models written in italics are starting models, models in bold the final ones (with significant variables)

Link	Model I	Model II	Model III
Probability of successful reproduction			
Logit	$habitat+\varepsilon_{t}$	habitat×year	$habitat+\varepsilon_{t}$
	$\varepsilon_{t}∼N\left(0,\sigma_{\mathrm{habitat}}\right)$	year	$\varepsilon_{t}∼N\left(0,\sigma_{\mathrm{habitat}}\right)$
Number of fledglings			
Identity	$habitat+\varepsilon_{t}$	habitat×year	$habitat\times \left(hat^{2}+cs+prec^{2}+temp^{2}+del+meth+partag\right)$
	$\varepsilon_{t}∼N\left(0,\sigma_{\mathrm{habitat}}\right)$	habitat + year	habitat x temp^2^ + hat^2^ + cs + prec^2^ + del
First‐year survival			
logit	$habitat+\varepsilon_{t}$	habitat×year	$prec^{2}+temp^{2}+del+meth+partag$
	$\varepsilon_{t}∼N\left(0,\sigma_{\mathrm{habitat}}\right)$	year	prec^2^ + temp + del
Adult survival			
Logit	$habitat+\varepsilon_{t}$	habitat×year	$prec^{2}+temp^{2}+tag$
	$\varepsilon_{t}∼N\left(0,\sigma_{\mathrm{habitat}}\right)$	1	temp
Immigration rate			
logit	$habitat+\varepsilon_{t}$	habitat×year	1
	$\varepsilon_{t}∼N\left(0,\sigma_{\mathrm{habitat}}\right)$	**1**	

In the first step, annual variation in habitat‐specific demographic rates was modeled using independent random effects of year (model I). Random effects of year can capture both the decline and the variability of the population dynamics. Thus, model I was used to investigate the contributions of habitat‐specific demographic rates to the dynamics of the complete population. We performed a retrospective analysis, following Koons et al. (2016). We estimated the contributions of each habitat‐specific demographic rate and of the population structure (number of individuals per stage class and per habitat quality level) to the temporal variation in the realized population growth rate ( $var\left(\lambda_{\mathrm{realized}}\right)$ ) and to the difference in the mean population growth rate ( $\Delta log\lambda_{g}$ ) between the three years with the strongest population increase (2002–2005) and the three years with the strongest population decline (2013–2016). As the movement probabilities showed weak variation among years, they were kept constant over years in the following models II and III.

In step II, we studied whether demographic rates showed spatiotemporal trends. The annual variation of each demographic rate in each level of habitat quality was modeled using a linear temporal trend (year as a continuous variable). For each demographic rate, we tested whether the demographic rates were declining and whether possible trends were heterogeneous among levels of habitat quality by inspecting whether the 95% credible intervals of the interactions between habitat quality and year included 0.

In step III, annual variation in habitat‐specific demographic rates was modeled using explanatory variables in order to identify possible drivers of the population dynamics (model III). We used weather variables and variables characterizing research activity (see below and Table 2).

**Table 2:** Description of explanatory variables

Name	Abbreviation	Type	Description
Weather			
Precipitation	Prec	Continuous variable	Annual sum of spring precipitation
Temperature	Temp	Continuous variable	Annual mean of spring temperature
Research activity			
Capture delay	Del	Threshold variable	Time elapsed between the capture date of the mother and the hatching date of the last egg
Capture method	Meth	Binary variable	Captured inside or outside the nest box
Tagging	Tag	Binary variable	Whether an individual was equipped with a tag (VHS, geolocators or GPS)
Parental tagging	Partag	Binary variable	Whether at least one of the parents was tagged during the reproductive season
Other			
Hatching date	Hat	Continuous variable	Hatching date of the first brood
Clutch size	Cs	Continuous variable	The number of eggs laid by a female during a season

Cool and rainy days in spring are known to influence negatively reproductive success in hoopoes (Arlettaz, Schaad, Reichlin, & Schaub, et al., 2010). Thus, we tested the influence of the sum of precipitation and mean temperature in spring in the study site on survival and reproductive rates. Because the best weather conditions are often at intermediate values, we included quadratic effects of spring precipitation and temperature.

We used different variables to test the influence of the research activity on survival and productivity. Variables of research activity included tagging (whether an individual carried a tag), the capture method (inside or outside of the nest box), and the capture delay (number of days elapsed between the capture of the mother and the hatching of the last egg). We tested the influence of tagging on adult survival because supplemental manipulation or additionally carrying weight might influence individual survival. We tested the influence of parental tagging, of the maternal capture method, and of maternal capture delay on first‐year survival and on the number of fledglings produced. Parental tagging may affect efficiency of foraging and can thus have consequences on feeding rates and on fledgling survival. We investigated the effect of the capture method of mothers because females captured inside the nest box (thus on the nest) are forced to interrupt brooding which may negatively affect the nestlings. We investigated the influence of maternal capture delay on the number of fledglings produced and on first‐year survival to test whether the delay between female capture and hatching of the last egg influenced more productivity than the capture method. Because we tested the influence of capture delay on total female productivity, we averaged capture time over broods for females that had more than one brood in a given year. The influence of the capture delay on the number of fledgling and on first‐year survival was modeled with a threshold effect. This model assumes that the number of fledglings produced and first‐year survival does not change when the capture delay was positive (when the mother was captured after the hatching of the last egg, regression slope is fixed to zero), but when the capture delay was negative, the relationships are estimated.

Finally, we included the simple effects of the number of eggs laid by a female in a year and hatching date of the first brood on the number of fledglings produced as these variables are known to influence reproductive success in hoopoes (Plard et al., 2018; Price, Kirkpatrick, & Arnold, 1988).

Hoopoes from our study site spend the nonbreeding season at very different locations in a vast area south of the Sahara desert extending from Senegal to Niger (van Wijk, Bauer, et al., 2016), but a previous study showed that the environmental condition during the nonbreeding season did not appear to influence the population performance (Souchay, Wijk, Schaub, & Bauer, 2018). Therefore, we did not consider environmental variables from the nonbreeding period.

We simplified the starting model III by successively removing the interactive effects between habitat quality and explanatory variables whose 95% credible intervals had the largest overlap with 0. Finally, we estimated the percentage of variation in population size explained by the covariables selected in the restricted model III (Gelman & Pardoe, 2006). We estimated the temporal variance explained by the complete model using the variance of the residuals over the variance of the observed population ( $R_{\mathrm{tot}}^{2}$ ). Then, we estimated the contribution of each selected variable to the variation in the observed population size by refitting the selected model III excluding successively each explanatory variable ( $\left[R_{\mathrm{tot}}^{2}-R_{-variable}^{2}\right]/R_{\mathrm{tot}}^{2}$ ). We also assessed the contribution of each of these covariables to the temporal trend observed in the population from 2005 onward by excluding successively each explaining variable and deducing the percentage of the trend explained by each variable ( $\left[trend_{\mathrm{tot}}-trend_{-variable}\right]/trend_{\mathrm{tot}}$ ).

## 3 RESULTS

**Figure 2:**
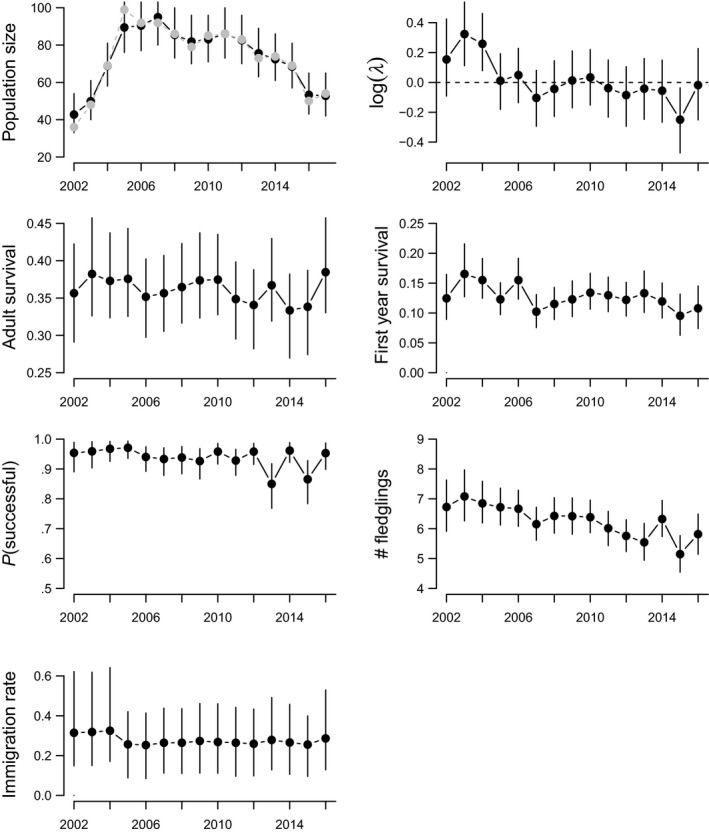
Population growth rate, population size, and demographic rates of the hoopoe population in the Valais, Switzerland, from 2002 to 2017. Estimates are predicted from an IPM that combined the three habitats and that included year as a random effect on each demographic rates. The predicted and observed population sizes are shown in black and gray, respectively.

The population first strongly increased and then steadily declined, but the population growth rate ( $log\left(\lambda \right)$ ) has declined from the beginning of the period under consideration here (2002–2017, Figure 2) and dropped below 0 (hence the population declined) after 2005. The increase in the population (i.e., population growth rate above 0) in the three first years was characterized by simultaneous and relatively high overall productivity, first‐year survival, and immigration (Figure 2).

**Figure 3:**
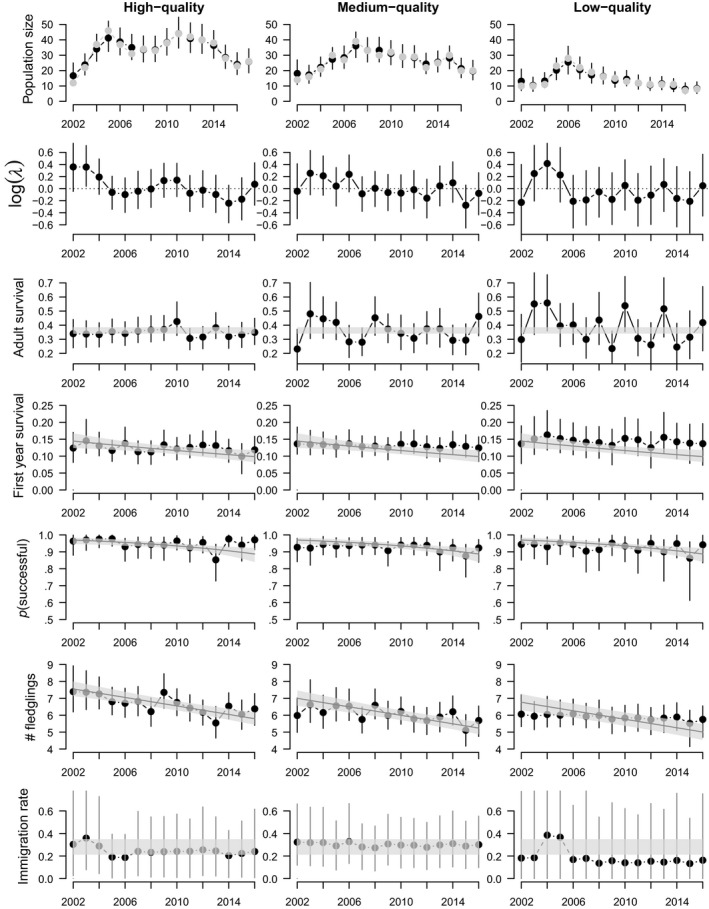
Population growth rate, population size, and demographic rates of the hoopoe population in the Valais, estimated with the spatial IPM stratified by habitat. Shown are estimates from Model I with years as random effects (points) and from Model II with years as continuous effects (lines when the 95% credible interval of the effect of year did not overlap with 0). Points and lines are posterior means, and the vertical lines and the gray layers show the 95% credible intervals. The observed population size is also shown by gray points.

Models I showed that temporal patterns in population sizes were similar in all habitats (Table 1 and Table S1) with first an increasing and later a decreasing period (Figure 3). First‐year individuals dispersed more often than adults between patches of different habitat quality (Table S1). Indeed, the majority of adults were faithful to their habitat type. If dispersal occurred, it was stronger toward a habitat of higher quality than to a habitat of lower quality (Table S1).

### 3.1 Contribution of demographic rates to population growth rate

**Figure 4:**
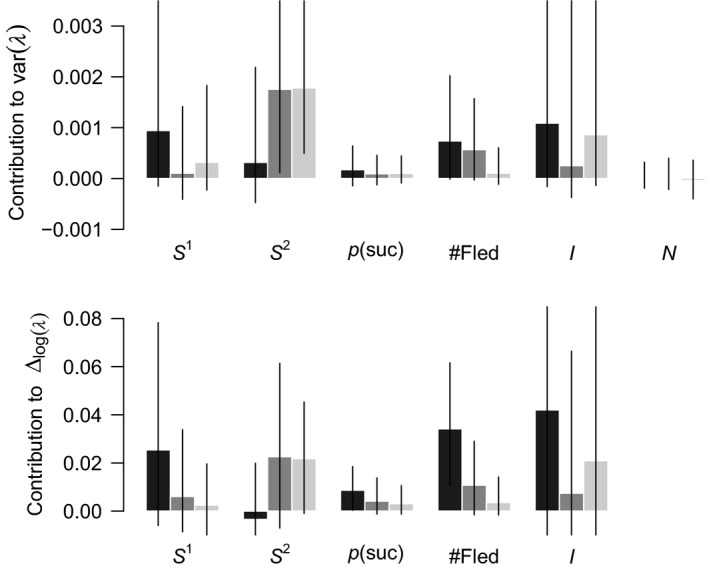
Contributions of demographic rates from the three habitats of different quality (high: black, medium: dark gray, low: light gray) to the temporal variation in the realized population growth rate ( $var\left(\lambda \right)$ ) and to the difference in mean population growth rate ( $\Delta log\left(\lambda \right)$ ) between 2002–2005 and 2013–2016. Contributions of first‐year and adult survival ( $S^{1}$ and $S^{2}$ ), probability of successful reproduction ( $P\left(\mathrm{suc}\right)$ ), number of fledglings (#Fled), immigration ( I ), and of population structure (N) are shown. Bars are posterior means, and the vertical lines show the 95% credible intervals. For the contribution to $\Delta log\left(\lambda \right)$ , only the mean direct effects from the difference in the mean of each demographic rate are shown (all other direct and indirect contributions can be found in Table S6).

The observed temporal variation in population growth rates was chiefly driven by the variation in the demographic rates while the variation in population structure (relative number of females among the different habitats and across age classes) contributed virtually nothing (Table S6). Variation in adult survival in habitats of medium and low quality and in the number of fledglings in high‐quality habitats were the only rates that contributed significantly to the variation in the population growth rate ( $Var\left(\lambda \right)$ , Figure 4). Although the contribution of first‐year survival in habitats of low and of adult survival in habitats of medium quality was the largest overall, the uncertainty was very pronounced, hampering inference (Figure 3). Changes in the demographic rates in high‐quality habitats contributed more to the decrease in λ (Figure 4; Table S6) between the first (2002–2005) and the second period (2013–2016) than that of the demographic rates in medium or low habitat quality. The overall strongest contribution was due to the decline of the number of fledglings in habitats of high quality.

### 3.2 Spatial heterogeneity in trends

Using model II, we did not find habitat‐specific heterogeneity in annual trends of the different demographic rates (Tables S2 and S3). The number of fledglings produced by successful birds and first‐year survival declined by 25%[18%:32%] and 31%[10%:48%], on average, respectively, over the study period (Figure 3). Adult survival showed a weak but not significant decrease (16%[−2%:34%]) and immigration remained almost constant after the three first years (Table S3; Figure 3).

The average number of fledglings increased with increasing habitat quality, being 5.9[5.4:6.3], 6.0[5.6:6.5], and 6.7[6.2:7.2] for habitats of low, medium, and high quality, respectively, in the first years of the study. Other demographic rates were not affected by habitat quality.

### 3.3 Impact of weather and research activity

**Figure 5:**
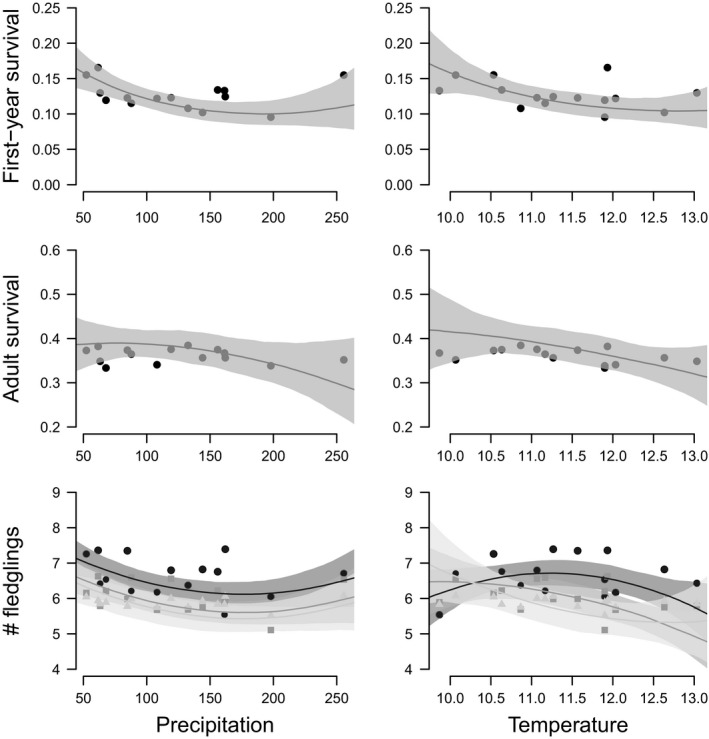
Influence of the sum of spring precipitation and mean spring temperature on first‐year survival, adult survival, and the number of fledglings produced by a successful female. Points and segments show the posterior means and 95% credible intervals of the demographic rates estimated from the model I including year as a random effect. The lines and gray shades show the posterior means and 95% credible intervals of the demographic rates estimated from the model III. For the number of fledglings produced, predictions from high, medium, and low‐quality habitats are shown in black, dark gray, and light gray, respectively.

Effects of spring temperature on productivity differed according to habitat quality (Table S4). While in high‐quality habitats the number of fledglings was highest at intermediate ambient temperature, it decreased with decreasing temperatures in habitats of low and medium quality (Table S5; Figure 5). First‐year and adult survival decreased in all habitats with decreasing ambient temperature (Table S5; Figure 4). The effect of spring precipitation on demographic rates was consistent among habitats. First‐year survival and the number of fledglings produced decreased with increasing spring precipitation (Table S5; Figure 5). A quadratic effect of spring precipitation on both demographic rates was significant because of the year 2006 that was a year of high performance but also of high precipitation (Table S5; Figure 5) but the quadratic effect is not really supported by the data.

Among the variables describing the research activity, only the capture delay had an impact on demography. It was positively related to the number of fledglings produced and with first‐year survival when the female was captured before the hatching of the last egg (Table S5; Figure S4).

Spring temperature and precipitation have slightly but not significantly increased over the study period (slope for standardized precipitation: $0.32\left[-5.10:5.74\right]$ , and standardized temperature: $0.05\left[-0.04:0.14\right]$ , Figure S3). Capture delay was the only variable that decreased significantly until 2015 ( $slope=-0.09\left[-0.14:-0.09\right]$ , Figure S2), but strongly increased thereafter due to a change in the field protocol. The simultaneous influences of capture delay and weather variables on each demographic rates explained 19% of the decrease in the population growth rate (Figure S3). The effects of spring temperature contributed more to this decline (49% of the variance explained by the model and 9% of the observed decline in the population growth rate) than spring precipitation (36% of the variance explained and 7% of the observed decline) and capture delay (15% of the variance explained and 3% of the observed decline, Figure 5).

## 4 DISCUSSION

**Figure 6:**
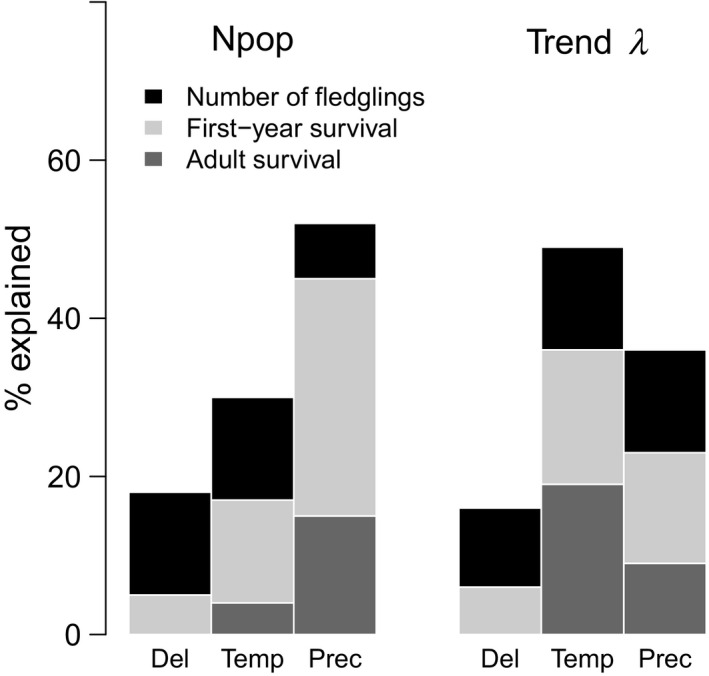
Percentage of variation in the population size (Npop) and in the slope of the decline in the population growth rate (trend) explained by the effects of the capture delay (Del) spring temperature (Temp) and spring precipitation (Prec).

The main demographic reasons for the decline of the studied hoopoe population were the decrease in the annual number of fledglings in successful broods and in apparent first‐year survival. Because a large proportion of the population was breeding in habitats of high quality, the overall population decline was especially due to the decrease in these demographic rates in habitats of the highest quality. Excluding immigration, variation in recruitment is often the main driver for the dynamics of small populations (Saether et al., 2016). However, assessing the role of the different demographic rates on weak and recent population declines remains difficult because of a lack of power to find significant correlations between population growth rate and demographic rates (Hayes & Steidl, 1997; Thogmartin et al., 2007). By estimating the relative contribution of each habitat‐specific demographic rate to the variation in the population growth rate (Koons et al., 2016), we identified the demographic rates responsible for the relatively recent decline of this hoopoe population (Figure 6).

The main driver for the observed reduction in productivity was a continuous decrease in fledging success that was not significantly different between habitats (Tables S2 and S3). Hoopoe females raised on average 7.3 [6.5:8.3] fledglings per year at the beginning of the study period, but only 5.6 [4.9:6.0] at the end. Thus, hoopoes seem to have more and more problems raising chicks, which may be due to a decline in the availability and/or quality of prey (driven by e.g., progressive climate shifts and/or unknown agricultural intensification processes), a direct impact of weather circumstances, and to a lower extent the pressure of research activities. Apparent first‐year survival also decreased by 31% over the study period. Although we cannot disentangle whether this stemmed from a decrease in true first‐year survival or from an increase in emigration, the decrease in both productivity and first‐year apparent survival certainly resulted in a magnified decrease in local recruitment, which contributed to the observed population decline.

### 4.1 Influence of habitat

Our integrated population model structured by habitat quality (i.e., prey availability) allowed us to determine the impact of habitat‐specific demography on recent population changes. Fledgling production was clearly different among habitats of different quality. Yet, the temporal trends were not significantly different between habitat qualities, suggesting that environmental conditions have similarly deteriorated in the whole study area. At this stage, one can only speculate about the possible mechanisms because our measure of habitat quality is only indirect, being based on the specificities of the ground water level. Indeed, ground water table is the best predictor of mole cricket abundance, the staple prey in our study hoopoe population, which affects their reproductive rate and territory occupancy (Guillod et al., 2016; Tschumi et al., 2014). While we know that hoopoes prefer foraging in sites with a great fraction of bare ground (Tagmann‐Ioset et al., 2012), this preference might be more related to prey accessibility than their occurrence and/or abundance. That is why we used the ground water table as a simple but seemingly powerful proxy of habitat quality, although a better assessment of hoopoe habitat quality would be welcome. As a matter of fact, marked changes in farming practices may have occurred during the 16 years study period in these intensively managed agroecosystems: in particular, more efficient but also more detrimental pesticides may have been applied, as observed elsewhere (Hallmann, Foppen, Turnhout, Kroon, & Jongejans, 2014), potentially impacting mole cricket abundance and hence pre‐ and postfledging survival as a consequence.

The IPM structured by habitat identified the demographic rates that contributed significantly to the variation of population growth, pointing to the key role played by habitat quality. The contribution of demographic rates to population growth depends on their elasticity and on their temporal variability (Heppell, Caswell, & Crowder, 2000; Saether & Bakke, 2000; Saether et al., 2016). Variation in apparent first‐year survival and in the number of fledglings produced by successful hoopoe females were the main mechanisms for the decline of the population growth rate. Processes in habitats of high quality were of particular importance for the decline of the population because productivity was largest, on average, in these habitats and most broods occurred there. Interestingly in habitats of lower quality, another demographic mechanism than local recruitment seemed to have driven population growth, namely apparent adult survival. A similar pattern was observed in a population of Wood thrushes (Rushing et al., 2017). However, given the large uncertainty around the estimates of apparent adult survival, this result should be taken with caution. Adult survival has evolved to be fairly stable in iteroparous species in general (Gaillard & Yoccoz, 2003) and this had been previously demonstrated for our hoopoe population as well (Schaub et al., 2012), pointing to a possible variation in emigration.

### 4.2 Impact of weather

We show that spring environmental conditions affected survival and productivity. The combined effects of weather variables on these demographic rates contributed to 16% of the decline in population growth rate. This relatively low contribution is most likely due to an absence of significant temporal trends in spring weather conditions. According to a previous study on the same population, prey biomass provisioned by parents to chicks as well as the proportion of mole crickets entering nestlings' diet is lower on rainy and cold days, affecting breeding success (Arlettaz, Schaad, Reichlin, & Schaub, 2010). If the present study confirms the positive effect of low rainfall on hoopoe productivity, it suggests that breeding success decreases with ambient temperature, which is in apparent contradiction to Arlettaz, et al., (2010). Yet, this discrepancy can be explained by different methodological approaches. Here, we used a single spring (March to May) ambient temperature mean for the total reproductive output of a female in a given year, irrespective of the timing of breeding, while in Arlettaz, Schaad, Reichlin, & Schaub, et al., (2010), the mean ambient temperature of reference was calculated for each brood separately, based on daily weather information for a narrow time window around the hatching period. The approach by Arlettaz, Schaad, Reichlin, & Schaub, et al., (2010) might therefore more accurately account for thermal conditions encountered during breeding.

### 4.3 Impact of research activity

We did not evidence any impact of tagging devices (geolocators, radio transmitters, and/or GPS) on individual performance in our study population in line with van Wijk, Souchay, Jenni‐Eiermann, Bauer, & Schaub (2016). In addition, the method used for capturing female parents (inside or outside the nest boxes) did not affect their breeding performance. In contrast, the timing of maternal capture in relation to brood development (capture delay) slightly but significantly affected both the number of fledglings raised and their first‐year survival. The progressive shortening of this delay over years (from 0.5 day *after* hatching of the last egg in the first study phase to 4.5 days *before* the hatching of the last egg, on average, in the second phase; Figure S2) has contributed to a reduction in annual productivity and hence population growth rate. In species with asynchronous hatching such as the hoopoe (Martín‐Vivaldi et al., 1999), it is important to delay the capture of the parents after the hatching date of the last egg in order to avoid reducing the survival of the youngest chicks that strongly rely on female brooding as long as they cannot thermoregulate by themselves, that is, up to 8–10 days after hatching (Ryser, Guillod, Bottini, Arlettaz, & Jacot, 2016). Otherwise, the low temperature may result in the death of some chicks or may negatively affect their development, reducing postfledgling survival. While the effects of tag deployment on individual behavior and performance are often investigated (Rasilius, Festa‐Bianchet, Couturier, & Côté, 2014; Saraux et al., 2011; van Wijk, Souchay, et al., 2016), the impact of research operations on demographic rates is either rarely considered or seldom reported. As soon as we found evidence for the negative impact of mother capture (2015), we changed our field protocol for the capture of breeding females such that mother captures were delayed at the earliest after hatching of the last egg. We have observed a slight increase in brood success (the probability of an egg to fledge increased from 0.66 in 2015 to 0.82 in 2016 and 2017) even if the population decline could not be halted.

### 4.4 Conservation implications

Despite the steep rise of the hoopoe study population following the sudden massive offer of artificial breeding sites near their main foraging grounds (Arlettaz, Schaub, et al., 2010), its productivity has declined, shallowly but significantly, over the years since the beginning of the period analyzed. The reason(s) for this progressive decline have long remained unknown, but this study provides first hints to orientate future conservation research and action. Although the rate of temporal decline has been similar across all three habitat qualities, our results suggest again the key role of prey, notably mole crickets, in productivity and survival. As established previously (Arlettaz, Schaub, et al., 2010; Guillod et al., 2016), reproductive success and fledglingsˊ body condition in our hoopoe population depend on the biomass and fraction of mole crickets entering nestlingsˊ diet, but the spatial distribution of this most profitable prey is heterogeneous across the study area (Tschumi et al., 2014). The spatial variation in prey availability will affect local demographic performance that can be achieved in different habitats (Blondel, Dias, Maistre, & Perret, 1993; Griffen & Norelli, 2015; Douglas, 1991; Penteriani, Mar Delgado, Gallardo, & Ferrer, 2004).

All in all, this suggests that prey abundance, prey availability (i.e., abundance modified by accessibility), notably of mole crickets, as well as food provisioning activity to chicks by parents may have decreased in recent time in the study area. Future research should thus focus on the spatiotemporal variability of mole cricket availability and on factors affecting it. This should include the sanitary quality of mole cricket prey, in particular in relation to pesticide application in agriculture. Until the situation of mole crickets is better understood, the only conservation measure we can suggest is to capture breeding female as late as possible during the chick rearing period. More generally, this study shows that integrated population models stratified by habitat are powerful tools to sort out what might be the main drivers beyond declines in small populations, allowing orienting research activity toward the most pressing conservation questions so as to develop sound conservation action.

## AUTHOR CONTRIBUTION

**Floriane Plard:** Conceptualization (equal); formal analysis (lead); methodology (lead); writing–original draft (lead); writing–review and editing (equal). **Raphaël Arlettaz:** Conceptualization (supporting); funding acquisition (equal); project administration (equal); writing–review and editing (equal). **Alain Jacot:** Conceptualization (supporting); funding acquisition (equal); project administration (equal); writing–review and editing (equal). **Michael Schaub:** Conceptualization (equal); funding acquisition (lead); project administration (lead); supervision (equal); writing–review and editing (equal). 

## COMPETING INTERESTS

We declare to have no competing interests.

## Supporting information

Supplementary Material

## Data Availability

The data sets are available in Dryad (https://doi.org/10.5061/dryad.xksn02vc3).
