# Supplementary material for: Disentangling the spatial and temporal causes of decline in a bird population
Source: Ecol Evol. 2020 Jun 30;10(14):6906–18. doi: 10.1002/ece3.6244 (PMC7391334; doi:10.1002/ece3.6244)
Supplement: Supplementary file 1 — Supplementary Material [file ECE3-10-6906-s001.pdf]

Disentangling the spatial and temporal causes of decline in a bird population

Floriane Plard[1,*], Raphaël Arlettaz[2,3], Alain Jacot[2,3], Michael Schaub[1]

1. Swiss Ornithological Institute, CH-6204 Sempach, Switzerland;

2. Division of Conservation Biology, Institute of Ecology and Evolution,University of Bern, Baltzerstrasse 6a, 3012 Bern, Switzerland.

3. Swiss Ornithological Institute, Valais Field Station, Rue du Rhône 11, 1950 Sion, Switzerland.

∗ Corresponding author; e-mail: floriane.plard@ens-lyon.fr

Online Supplementary material

Table S1: Mean and temporal variability of the demographic rates (**Model I** including year as a random effect). Posterior means and 95% credible intervals of each habitat-specific demographic rates are shown on the natural scale for mean and on the original scale for temporal variability (σ_t). γ corresponds to the second parameter of the gamma function for the annual number of fledglings produced per female.

habitat	**all**			**High-quality**			**Medium-quality**			**Low-quality**		
Probability of successful reproduction												
	mean	2.5%	97.5%	*mean*	2.5%	97.5%	*mean*	2.5%	97.5%	*mean*	2.5%	97.5%
mean	0.951	0.922	0.971	0.965	0.929	0.990	0.935	0.898	0.965	0.947	0.893	0.985
σ_t	0.808	0.395	1.421	1.072	0.244	2.451	0.532	0.032	1.269	0.826	0.053	2.466
Number of fledglings per female and year												
	mean	2.5%	97.5%	*mean*	2.5%	97.5%	*mean*	2.5%	97.5%	*mean*	2.5%	97.5%
mean	4.187	3.816	4.596	4.519	4.042	5.052	4.100	3.683	4.560	3.988	3.553	4.439
σ_t	0.433	0.233	0.704	0.504	0.164	0.925	0.425	0.078	0.825	0.242	0.001	0.673
γ	0.669	0.612	0.725	0.678	0.619	0.741	0.678	0.619	0.741	0.678	0.619	0.741
First-year survival												
	mean	2.5%	97.5%	*mean*	2.5%	97.5%	*mean*	2.5%	97.5%	*mean*	2.5%	97.5%
mean	0.125	0.107	0.144	0.122	0.102	0.143	0.130	0.110	0.151	0.142	0.113	0.174
σ_t	0.246	0.097	0.436	0.199	0.014	0.500	0.117	0.006	0.342	0.203	0.016	0.545
Adult survival												
	mean	2.5%	97.5%	*mean*	2.5%	97.5%	*mean*	2.5%	97.5%	*mean*	2.5%	97.5%
mean	0.361	0.332	0.393	0.347	0.302	0.397	0.353	0.283	0.432	0.374	0.277	0.491
σ_t	0.136	0.012	0.322	0.233	0.020	0.508	0.494	0.143	0.940	0.702	0.324	1.238
Immigration rate												
	mean	2.5%	97.5%	*mean*	2.5%	97.5%	*mean*	2.5%	97.5%	*mean*	2.5%	97.5%
mean	0.267	0.176	0.365	0.206	0.050	0.365	0.287	0.152	0.428	0.014	0.000	0.360
σ_t	0.404	0.017	1.292	0.905	0.029	4.002	0.445	0.016	1.476	2.766	0.033	9.382
Natal dispersal between habitats												
	mean	2.5%	97.5%	*mean*	2.5%	97.5%	*mean*	2.5%	97.5%	*mean*	2.5%	97.5%
$\Psi^1_{H\rightarrow}$				0.557	0.384	0.707	0.311	0.162	0.489	0.129	0.114	0.222
σ_t				0.532	0.056	1.230	0.787	0.162	1.651			
$\Psi^1_{M\rightarrow}$				0.412	0.337	0.479	0.357	0.300	0.443	0.218	0.203	0.246
σ_t				0.318	0.015	0.873	0.333	0.019	0.908			
$\Psi^1_{L\rightarrow}$				0.367	0.278	0.389	0.318	0.272	0.418	0.316	0.303	0.352
σ_t				0.311	0.009	0.975	0.459	0.034	1.247			
Breeding dispersal between habitats												
	mean	2.5%	97.5%	*mean*	2.5%	97.5%	*mean*	2.5%	97.5%	*mean*	2.5%	97.5%
$\Psi^2_{H\rightarrow}$				0.805	0.760	0.853	0.176	0.130	0.219	0.019	0.017	0.021
σ_t				0.253	0.006	0.709	0.294	0.015	0.799			
$\Psi^2_{M\rightarrow}$				0.301	0.245	0.335	0.569	0.525	0.618	0.136	0.127	0.143
σ_t				0.294	0.021	0.804	0.220	0.012	0.701			
$\Psi^2_{L\rightarrow}$				0.018	0.012	0.207	0.273	0.234	0.330	0.683	0.553	0.733
σ_t				3.972	0.466	9.408	0.367	0.013	1.123			
Recapture probabilities												
	mean	2.5%	97.5%	*mean*	2.5%	97.5%	*mean*	2.5%	97.5%	*mean*	2.5%	97.5%
$p(1yo)$	0.662	0.591	0.726	0.648	0.574	0.715	0.648	0.574	0.715	0.648	0.574	0.715
$p(>1yo)$	0.814	0.757	0.864	0.805	0.743	0.857	0.805	0.743	0.857	0.805	0.743	0.857
σ_t	0.309	0.019	0.660	0.328	0.042	0.663	0.328	0.042	0.663	0.328	0.042	0.663

Table S2: Assessing spatial heterogeneity in annual trends using **Model II**. Test of the spatial heterogeneity in the trends (slope) and in intercepts (int) of each demographic rate. Posterior means and 95% credible intervals of differences between the effects on each demographic rate in the three habitats are shown.

		High vs. Medium			High vs. Low			Low vs. Medium		
		mean	2.5%	97.5.5%	*mean*	2.5%	97.5.5%	*mean*	2.5%	97.5%
P(successful)	*slope*	0.035	−0.109	0.189	0.024	−0.158	0.203	0.011	−0.160	0.187
	int	0.312	−0.240	0.889	0.390	−0.288	1.048	−0.078	−0.711	0.599
# Fledglings	*slope*	−0.002	−0.065	0.064	0.018	−0.059	0.096	−0.020	−0.101	0.061
	int	0.371	0.113	0.654	0.530	0.223	0.859	−0.159	−0.481	0.167
1^{st}y Survival	*slope*	0.004	−0.047	0.057	0.015	−0.053	0.083	−0.011	−0.079	0.058
	int	−0.005	−0.205	0.190	−0.154	−0.388	0.078	0.148	−0.105	0.390
Ad. Survival	*slope*	−0.009	−0.064	0.045	0.029	−0.036	0.098	−0.038	−0.110	0.032
	int	−0.150	−0.364	0.056	−0.077	−0.328	0.178	−0.073	−0.339	0.206
Immigration	*slope*	−0.017	−0.189	0.180	6.787	−0.007	24.197	−6.804	−24.145	−0.026
	int	−0.221	−0.930	0.501	0.230	−0.777	1.630	−0.451	−1.857	0.553

Table S3: Posterior means, standard deviations (SD) and 95% credible intervals of parameters from **model II** that included a continuous effect of year on the demographic rates. The labels of habitat qualities are H (high), M (medium) and L (low). γ corresponds to the second parameter of the gamma function for the annual number of fledglings produced per female.

Probability of successful reproduction (logit scale)				
	mean	*SD*	2.5%	97.5%
intercept	3.578	0.350	2.912	4.286
year	−0.101	0.033	−0.167	−0.036
Number of fledglings (natural scale)				
	mean	*SD*	2.5%	97.5%
$intercept(H)$	5.165	0.253	4.662	5.647
$intercept(M)$	4.792	0.248	4.303	5.274
$intercept(L)$	4.635	0.251	4.134	5.142
year	−0.085	0.014	−0.113	−0.057
γ	0.673	0.030	0.615	0.733
First-year survival (logit scale)				
	mean	*SD*	2.5%	97.5%
intercept	−1.747	0.099	−1.941	−1.551
year	−0.031	0.011	−0.053	−0.009
Adult survival (logit scale)				
	mean	*SD*	2.5%	97.5%
intercept	−0.564	0.051	−0.662	−0.465
year	−0.019	0.011	−0.042	0.003
Immigration rate (logit scale)				
	mean	*SD*	2.5%	97.5%
intercept	−0.950	0.176	−1.312	−0.623
year	−0.009	0.056	−0.117	0.110
Recapture probabilities (logit scale)				
	mean	*SD*	2.5%	97.5%
$p(1yo)$	0.660	0.171	0.315	1.005
$p(>1yo)$	1.518	0.192	1.158	1.913
σ_t	0.410	0.156	0.146	0.769
Natal dispersal between habitats (natural scale)				
	mean	*SD*	2.5%	97.5%
$\Psi^1_{H \to H}$	0.526	0.029	0.469	0.583
$\Psi^1_{M \to H}$	0.415	0.034	0.349	0.482
$\Psi^1_{L \to H}$	0.344	0.044	0.261	0.433
$\Psi^1_{H \to M}$	0.339	0.028	0.285	0.394
$\Psi^1_{M \to M}$	0.367	0.034	0.301	0.435
$\Psi^1_{L \to M}$	0.363	0.047	0.274	0.458
$\Psi^1_{H \to L}$	0.135	0.020	0.098	0.177
$\Psi^1_{M \to L}$	0.217	0.029	0.163	0.276
$\Psi^1_{L \to L}$	0.293	0.042	0.215	0.377
Breeding dispersal between habitats (natural scale)				
	mean	*SD*	2.5%	97.5%
$\Psi^2_{H \to H}$	0.816	0.022	0.770	0.858
$\Psi^2_{M \to H}$	0.274	0.027	0.223	0.329
$\Psi^2_{L \to H}$	0.055	0.019	0.023	0.098
$\Psi^2_{H \to M}$	0.164	0.021	0.122	0.207
$\Psi^2_{M \to M}$	0.593	0.031	0.531	0.652
$\Psi^2_{L \to M}$	0.264	0.037	0.193	0.337
$\Psi^2_{H \to L}$	0.021	0.008	0.007	0.040
$\Psi^2_{M \to L}$	0.133	0.021	0.093	0.178
$\Psi^2_{L \to L}$	0.682	0.039	0.603	0.755

Table S4: Assessing spatial heterogeneity in the number of fledglings using **model III**. Test of spatial heterogeneity in the effect of the weather (precipitation and temperature), the research activity (tagging, capture method, capture delay), clutch size and hatching date on the number of fledglings raised by a successful female. Posterior means and 95% credible intervals of differences between habitats are shown.

	Low vs. High			Low vs. Medium			Medium vs.High		
	mean	2.5%	97.5%	*mean*	2.5%	97.5%	*mean*	2.5%	97.5%
cutch size	0.020	−0.418	0.421	−0.003	−0.453	0.417	0.023	−0.308	0.387
hatching date	0.167	−0.196	0.514	0.255	−0.080	0.625	−0.088	−0.364	0.185
hatching date^2	−0.060	−0.372	0.248	−0.038	−0.345	0.250	−0.023	−0.231	0.184
temperature	−0.227	−0.584	0.111	0.053	−0.319	0.406	−0.279	−0.563	0.016
temperature^2	0.365	0.026	0.705	0.235	−0.125	0.605	0.129	−0.152	0.425
precipitation	0.061	−0.353	0.462	0.017	−0.419	0.431	0.044	−0.301	0.404
precipitation^2	0.013	−0.288	0.331	0.018	−0.304	0.320	−0.005	−0.290	0.274
capture delay	−0.006	−0.848	0.835	−0.187	−1.151	0.756	0.181	−0.441	0.810
capture method	−0.395	−2.546	1.958	0.660	−1.616	3.136	−1.056	−2.829	0.608
parental tagging	−0.052	−0.481	0.390	−0.238	−0.674	0.210	0.186	−0.182	0.544
intercept	−0.935	−1.446	−0.422	−0.355	−0.891	0.183	−0.581	−1.019	−0.166

Table S5: Posterior means, standard deviations (SD) and 95% credible intervals of parameters from **model III** that included the impact of weather (precipitation and temperature), research activity (tagging, capture method, capture delay), and individual variables (hatching date, clutch size) on the demographic rates. γ corresponds to the second parameter of the gamma function for the annual number of fledglings produced per female.

Probability of successful reproduction (logit scale)				
	mean	*SD*	2.5%	97.5%
intercept	2.830	0.201	2.489	3.270
$\sigma_t H$	0.835	0.466	0.092	1.935
$\sigma_t M$	0.603	0.285	0.147	1.228
$\sigma_t L$	0.752	0.490	0.058	1.878
**Number of fledglings (natural scale)**				
	mean	*SD*	2.5%	97.5%
InterceptH	5.761	0.281	5.222	6.296
InterceptM	5.180	0.260	4.696	5.686
InterceptL	4.826	0.294	4.259	5.418
clutchsize	0.268	0.077	0.124	0.423
$hatching\ date^2$	0.111	0.053	0.007	0.216
hatching date	−1.014	0.097	−1.207	−0.824
$temperature^2 H$	−0.237	0.104	−0.440	−0.022
$temperature^2 M$	−0.107	0.109	−0.324	0.097
$temperature^2 L$	0.128	0.158	−0.171	0.441
temperatureH	−0.124	0.105	−0.324	0.081
temperatureM	−0.403	0.115	−0.630	−0.174
temperatureL	−0.350	0.158	−0.654	−0.046
$precipitation^2$	0.146	0.059	0.027	0.260
precipitation	−0.287	0.091	−0.484	−0.124
capture delay	0.464	0.135	0.186	0.716
capture method	0.828	0.508	−0.113	1.882
parental tagging	−0.051	0.085	−0.217	0.119
γ	0.862	0.037	0.787	0.933
**First-year survival (logit scale)**				
	mean	*SD*	2.5%	97.5%
intercept	−2.104	0.090	−2.273	−1.921
$temperature^2$	0.056	0.048	−0.045	0.150
temperature	−0.146	0.049	−0.240	−0.052
$precipitation^2$	0.073	0.038	0.003	0.151
precipitation	−0.191	0.056	−0.307	−0.086
capture delay	0.016	0.006	0.003	0.029
capture method	0.016	0.313	−0.647	0.588
parental tagging	−0.013	0.092	−0.192	0.175
**Adult survival (logit scale)**				
	mean	*SD*	2.5%	97.5%
intercept	−0.447	0.087	−0.618	−0.285
$temperature^2$	−0.024	0.048	−0.119	0.068
temperature	−0.124	0.057	−0.229	−0.012
$precipitation^2$	−0.036	0.045	−0.123	0.047
precipitation	−0.068	0.064	−0.190	0.064
tagging	−0.168	0.104	−0.385	0.031
**Immigration rate (logit scale)**				
	mean	*SD*	2.5%	97.5%
intercept	0.278	0.037	0.206	0.355
**Recapture probabilities (logit scale)**				
	mean	*SD*	2.5%	97.5%
$p(1yo)$	0.701	0.153	0.402	1.012
$p(>1yo)$	1.482	0.169	1.152	1.801
σ_t	0.306	0.174	0.017	0.672

Table S6: Contribution of the habitat-specific demographic rates to $\Delta log(\lambda)$. For each demographic rate, we show direct contributions through variation in its mean (μ_D) and its variance (σ^2_D) and indirect contributions through variation in the means (μ_N) and the variances (σ^2_N) of the population structure.

	rate	μ_N	σ^2_N	μ_D	σ^2_D
High-quality	$\Psi^1_{\to H}$	$-6.879E-04$	$-1.812E-04$	0	$-1.812E-04$
	$\Psi^1_{\to M}$	$2.391E-04$	$2.887E-04$	0	$2.887E-04$
	$\Psi^2_{\to H}$	0	$-4.795E-06$	0	$-4.795E-06$
	$\Psi^2_{\to M}$	$2.281E-07$	$8.373E-06$	0	$8.373E-06$
	First-year survival	$-7.458E-05$	$7.286E-06$	$2.544E-02$	$7.286E-06$
	Adult survival	$4.957E-05$	$-5.916E-05$	$-3.454E-03$	$-5.916E-05$
	P(successful)	$-2.658E-05$	$-6.610E-06$	$8.678E-03$	$-6.610E-06$
	# fledglings	$-1.040E-04$	$3.604E-06$	$3.421E-02$	$3.604E-06$
	Immigration	$-9.613E-04$	$-5.109E-05$	$4.205E-02$	$-5.109E-05$
Medium-quality	$\Psi^1_{\to H}$	$-5.958E-05$	$-9.010E-05$	0	$-9.010E-05$
	$\Psi^1_{\to M}$	$-7.665E-07$	$-4.435E-06$	0	$-4.435E-06$
	$\Psi^2_{\to H}$	0	$-3.278E-05$	0	$-3.278E-05$
	$\Psi^2_{\to M}$	$1.803E-05$	$3.300E-05$	0	$3.300E-05$
	First-year survival	$6.940E-06$	$-1.090E-06$	$6.082E-03$	$-1.090E-06$
	Adult survival	$1.668E-04$	$-9.477E-05$	$2.261E-02$	$-9.477E-05$
	P(successful)	$4.773E-06$	$-5.263E-07$	$4.179E-03$	$-5.263E-07$
	# fledglings	$1.188E-05$	$-2.831E-06$	$1.078E-02$	$-2.831E-06$
	Immigration	$1.554E-04$	$-1.774E-05$	$7.445E-03$	$-1.774E-05$
Low-quality	$\Psi^1_{\to H}$	$-3.421E-06$	$-1.352E-04$	0	$-1.352E-04$
	$\Psi^1_{\to M}$	$-1.412E-06$	$-1.125E-04$	0	$-1.125E-04$
	$\Psi^2_{\to H}$	0	$7.292E-04$	0	$7.292E-04$
	$\Psi^2_{\to M}$	$-8.877E-07$	$6.367E-04$	0	$6.367E-04$
	First-year survival	$1.840E-05$	$-1.653E-06$	$2.516E-03$	$-1.653E-06$
	Adult survival	$3.052E-04$	$-2.748E-06$	$2.180E-02$	$-2.748E-06$
	P(successful)	$2.199E-05$	$-2.051E-05$	$3.098E-03$	$-2.051E-05$
	# fledglings	$2.579E-05$	$-3.974E-06$	$3.573E-03$	$-3.974E-06$
	Immigration	$4.327E-04$	$-1.918E-04$	$2.094E-02$	$-1.918E-04$

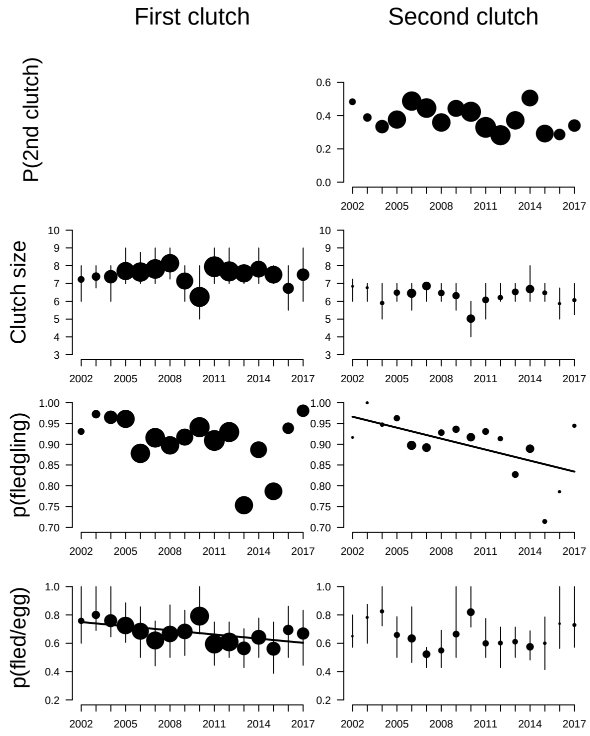

Figure S1: Decomposition of average annual reproductive success per female according to the probability of double-breeding (p(2nd clutch)), the mean clutch size, the annual probability of a clutch to produce at least one fledgling (p(fledgling)), and the annual probability of an egg belonging to a successful brood to become a fledgling (p(fled/egg)) for first and second broods. The size of the symbols is proportional to sample size.

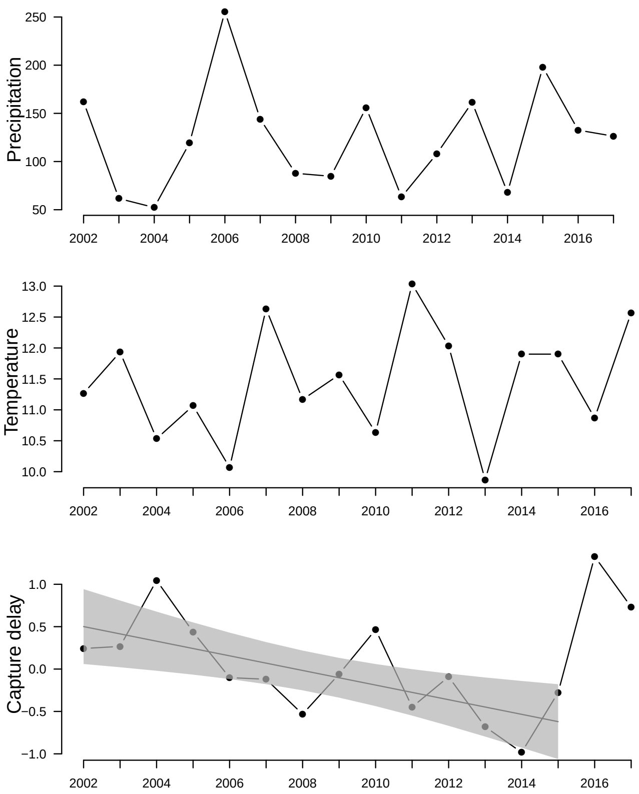

Figure S2: Annual estimates of the sum of spring precipitation, the mean spring temperature and the mean delay in maternal capture date. Year has a significant continuous effect only on the delay between hatching date of the last egg and maternal capture date when the last 2 years are not included.

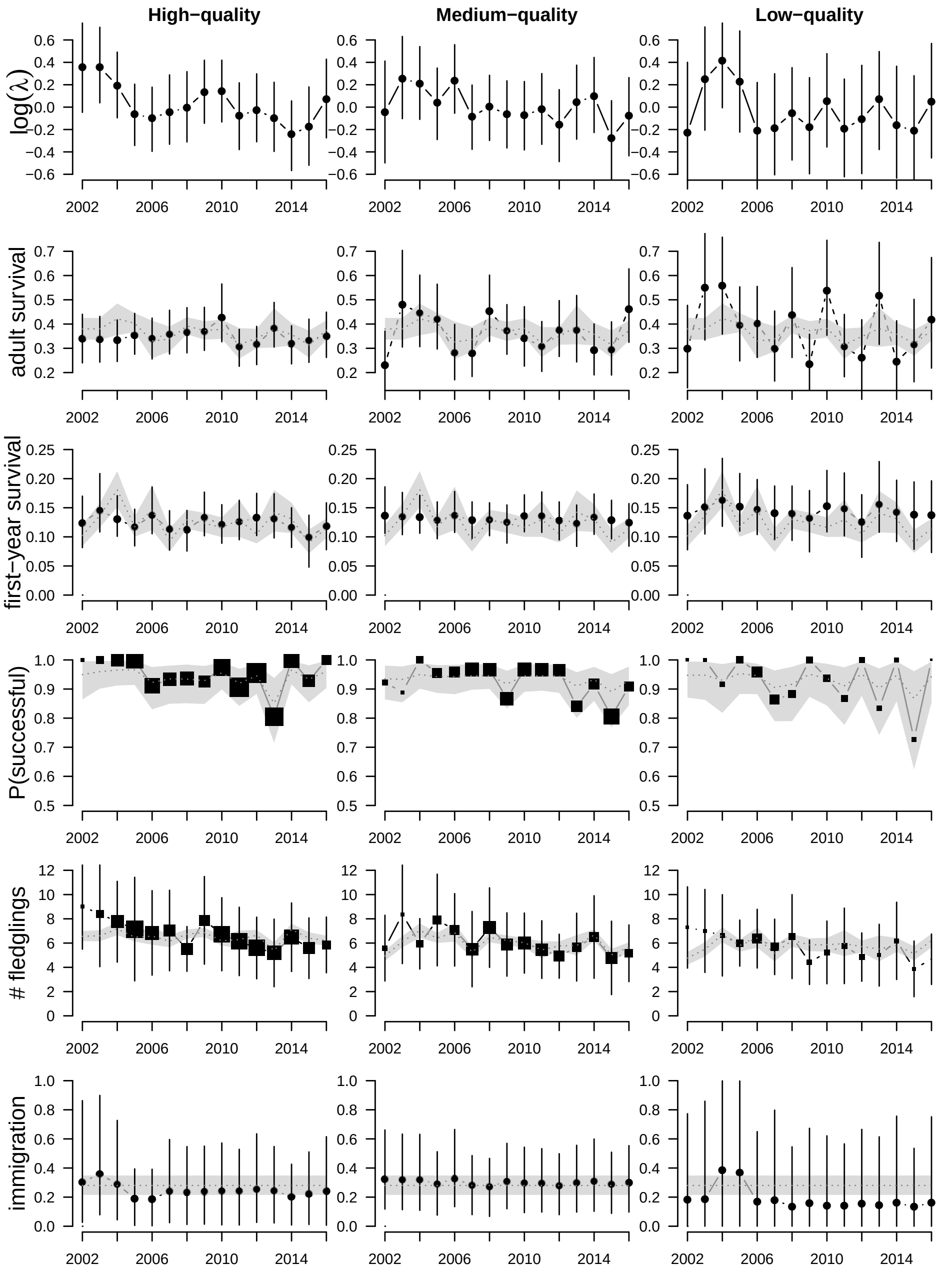

Figure S3: Impact of weather and individual variables on the habitat-specific demographic rates. Posterior means and 95% credible intervals of habitat-specific demographic estimated from Model III including weather and individual variables are shown by dotted lines and grey shades. Black points show the estimates from the model I including the influence of year as a random effect on each habitat-specific demographic rates. Black squares show the empirical reproductive data and their size is proportional to the number of females they represent.

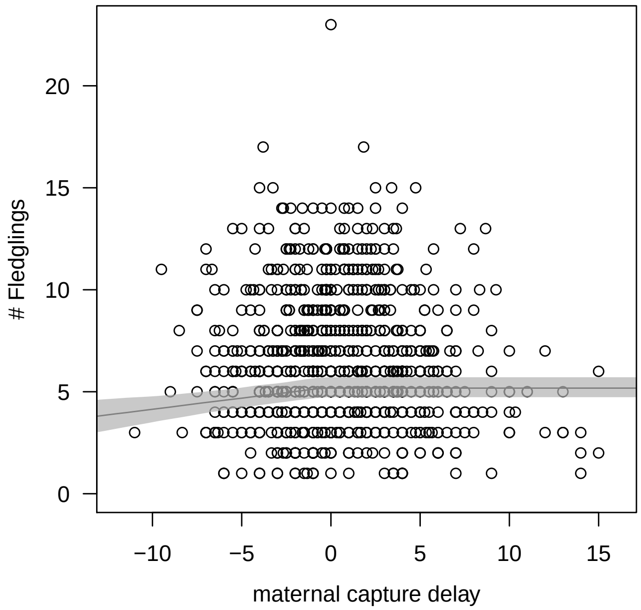

Figure S4: Influence of the delay between hatching date of the last egg and maternal capture date on the number of fledglings produced by a successful female. Maternal capture time can be negative if a female has been captured before all eggs had hatched.
